# Divergent demographic responses of boreal-breeding ducks to growing season variability

**DOI:** 10.1007/s00442-026-05865-x

**Published:** 2026-02-04

**Authors:** David J. Messmer, Stuart Slattery, Mark C. Drever, Chris Derksen, Robert G. Clark

**Affiliations:** 1 Department of Biology, 112 Science Place, University of Saskatchewan, Saskatoon, SK S7N 2E5 Canada; 2 Ducks Unlimited Canada, Institute for Wetland and Waterfowl Research, PO Box 1160, Stonewall, MB R0C 2Z0 Canada; 3 Pacific Wildlife Research Centre, Environment & Climate Change Canada, 5421 Robertson Road, Delta, BC V4K 3N2 Canada; 4 Climate Processes Section, Environment & Climate Change Canada, 4905 Dufferin Street, Toronto, ON M3H 5T4 Canada; 5 Prairie and Northern Wildlife Research Centre, Environment & Climate Change Canada, Saskatoon, SK S7N 0X4 Canada

**Keywords:** Western boreal forest, Normalized difference vegetation index, Population ecology, Spring phenology

## Abstract

In seasonal environments climatic variability shapes the timing, duration, and magnitude of primary production with effects that may percolate upward through food chains. While the role of spring phenology and implications of trophic mismatches have been documented in some bird species, including waterfowl, there is little research on the role of growing season duration or overall productivity. Duck species breeding in the western boreal forest (WBF) of North America vary widely in their average timing and plasticity for breeding dates, which may set up differing sensitivity to spring phenology. In contrast, increases in growing season duration and productivity may positively impact species regardless of life history through the extension of the breeding season and bottom-up trophic enrichment. We tested these hypotheses using breeding population estimates for 8 species (or species groups) of ducks and normalized difference vegetation index to infer spring phenology, length of growing season, and growing season productivity for duck survey areas in the WBF, 1982–2019. Spring phenology had mixed effects on species’ population growth rates, and effects were generally not consistent with mediation by average breeding dates or plasticity. Length of season and productivity effects showed some species’ population growth rates decreasing following longer seasons or higher productivity, opposite our predictions. These results suggest that, for secondary consumers like ducks, the role of growing season characteristics may be mediated or superseded by more complex biotic and abiotic interactions than can be explained with simple annual summaries of growing season characteristics.

**Supplementary Information:**

The online version contains supplementary material available at 10.1007/s00442-026-05865-x.

## Introduction

Climatic variability can drive substantial fluctuations in animal populations by influencing reproduction, survival, and dispersal (Lande et al. 2003; Knape and de Valpine 2011). In highly seasonal environments, such as northern breeding areas, interannual variation in timing, duration, and productivity of the growing season may generate bottom-up effects that propagate through food webs (Sæther et al. 2004; Mysterud et al. 2008; Fernández et al. 2016). These fluctuations may influence the timing and abundance of food resources and vegetative cover critical for reproduction. Across the Northern Hemisphere, climate warming has altered multiple dimensions of the growing season, including its onset, length, and overall productivity (Barichivich et al. 2013; Forkel et al. 2016; Piao et al. 2019). Such changes can modify the timing and extent of resource availability for migratory birds that depend on short, productive breeding seasons. Consequently, growing season variability may shape demographic outcomes, including annual population growth rates, and its effects may depend on species’ life-history traits and their capacity for behavioral or phenological plasticity (Charmantier et al. 2008; Visser and Gienapp 2019).

One of the most conspicuous and widely studied aspects of growing season change is the earlier onset of spring, reflected in advancing snow and ice melt, vegetation green-up, and prey emergence. Earlier springs have been widely documented across northern ecosystems (Barichivich et al. 2013; Ovaskainen et al. 2013), with consequences for migratory timing, reproductive schedules, and demographic rates in a range of taxa (Crick 2004; Møller et al. 2008; Jones and Cresswell 2010; Iler et al. 2021; Romano et al. 2023). The mechanisms linking phenological shifts to avian population dynamics are diverse. Effects may be direct, such as weather-mediated mortality during sensitive life stages or indirect, such as trophic mismatches between peak food availability and breeding activity (Hansson et al. 2014), or climate-driven changes in habitat structure (Foden et al. 2013). Numerous studies have documented negative fitness consequences when phenology of breeding or migration becomes decoupled from resource peaks (Visser et al. 1998; Both et al. 2006; Saino et al. 2011), including in some waterfowl (Brook et al. 2015; Ross et al. 2017, 2018). However, in some contexts, earlier springs have been associated with higher nesting success or longer breeding windows (Weiser et al. 2017). The degree and direction of these impacts may depend on traits such as average breeding dates and plasticity to adjust breeding to phenological cues (Charmantier et al. 2008; Verhulst and Nilsson 2008; Visser and Gienapp 2019; Messmer et al. 2021).

Beyond phenological timing, climate-induced changes in the length and cumulative productivity of the growing season may also affect breeding success. Across much of the Northern Hemisphere, growing seasons have lengthened in recent decades (Barichivich et al. 2013; Wang et al. 2016; Xiang et al. 2024), potentially allowing for extended breeding opportunities for birds, particularly via renesting after initial clutch failure (Halupka and Halupka 2017; Halupka et al. 2021). Additionally, higher cumulative photosynthetic productivity may enhance demographic performance through bottom-up food web enrichment (Scherber et al. 2010; Shurin et al. 2012; Gilson and McQuaid 2023). While such effects have been documented in primary consumers (Andreo et al. 2009; Pettorelli et al. 2009; Kanaziz et al. 2022), empirical studies evaluating the impact on higher trophic-level consumers like breeding birds remain scarce. Moreover, any increases in reproductive output arising from favorable growing season conditions may be dampened by density-dependent processes operating later in the annual cycle (Reed et al. 2013).

Ducks (*Anatinae*) represent a diverse group of secondary consumers that exhibit species-specific sensitivity to climate-driven environmental variation and change due to differences in life-history strategies. For instance, early breeders such as mallards (*Anas platyrhynchos*) and common goldeneye (*Bucephala clangula*) initiate nesting soon after ice melt and show high plasticity in responding to spring phenology, whereas late breeders like scaup and scoter may delay nesting for weeks and are less responsive to spring phenology cues (Toft et al. 1984; Gurney et al. 2011; Drever et al. 2012; Raquel et al. 2016; Messmer et al. 2021). Early breeders may benefit from earlier springs through better temporal alignment with food availability and greater renesting opportunities (Clark et al. 2014), or else simply have no response because they are able to track changes sufficiently. In contrast, late breeders may be at risk of phenological mismatch if peaks in key prey resources advance without a corresponding shift in nesting (Drever et al. 2012; Ross et al. 2015).

The western boreal forest (WBF) of Canada is a region of continental importance for breeding ducks (Slattery et al. 2011). It contains abundant wetlands embedded in a forested landscape (Gingras et al. 2016) and, despite relatively low direct human disturbance (but see Foote and Krogman 2006), faces an increasing risk from climate change. To evaluate potential climate impacts, we inferred growing season characteristics using remotely sensed normalized difference vegetation index (NDVI), a measure of vegetation "greenness" that reflects snow and ice cover, and the intensity of photosynthetic activity in northern latitudes (Myneni et al. 1997; Shabanov et al. 2002; Dye and Tucker 2003; Stöckli and Vidale 2004; Khare et al. 2019), and has seen increasing use in animal ecology (Pettorelli et al. 2011). While NDVI primarily captures terrestrial phenology, it also reflects climatic conditions (e.g., temperature and precipitation; Barichivich et al. 2013) that directly influence aquatic system phenology. Additionally, terrestrial photosynthetic productivity may serve as a proxy for overall summer temperatures and precipitation levels, with lower productivity typically associated with cooler temperatures or drought conditions (Peters et al. 2002; Maselli et al. 2003).

We assessed the relationship between interannual variation in NDVI-inferred growing season characteristics and annual population growth rates for eight boreal-breeding duck species or species groups. These species vary in average nest initiation timing and flexibility (Table 1; Gurney et al. 2011; Drever et al. 2012; Raquel et al. 2016; Messmer et al. 2021), which may mediate responses. Specifically, we tested three hypotheses. First, the phenological mismatch hypothesis proposes that species that nest early- to mid-season would exhibit no response or a positive response to earlier spring phenology as these species adjust their breeding dates to spring phenology, while late-nesting species would respond negatively due to potential trophic mismatches. Second, the season length hypothesis asserts that longer growing seasons would positively impact annual population growth across all species by providing extended time for renesting, duckling growth, and nutrient acquisition before fall migration. Third, the cumulative productivity hypothesis predicts that higher seasonal productivity would increase population growth rates across all species through improved food chain productivity and as a proxy for favorable environmental (non-drought) conditions.

**Table 1:** Breeding characteristics of duck species (or species groups) breeding in the western boreal forest study area

Species	Timing of clutch initiation	Renesting propensity	Diet during breeding season	Primary wintering area
Mallard	Late April, May, June	Commonly re-nests if first clutch is lost	Aquatic insects and invertebrates	Central US States
Goldeneye	Late April, May	Unlikely to re-nest after clutch loss	Aquatic insects and invertebrates (esp. molluscs)	Atlantic and Pacific coasts, large interior waterbodies of US and Canada
Bufflehead	Late April, May	Unlikely to re-nest after clutch loss	Aquatic insects and invertebrates	Atlantic, Pacific and Gulf coasts, lower Great Lakes
Green-winged teal	May, June	Can re-nest if first clutch lost	Aquatic insects and invertebrates; seeds	Pacific, Atlantic and Gulf coasts, Interior US and Mexico
Ring-necked duck	May, June	Can re-nest if first clutch lost	Aquatic insects and invertebrates; seeds	Atlantic coast south of Massachusetts, East Central US states
American Wigeon	June	Can re-nest if first clutch lost	Aquatic insects and invertebrates; seeds and fruits	US Pacific Northwest, California, Atlantic and Gulf Coast US States south of Massachusetts
Scaup	June	Unlikely to re-nest after clutch loss	Aquatic insects and invertebrates (esp. amphipods, molluscs), seeds	US Atlantic and Gulf Coasts south of Maine, including Mexico
Scoter	June	Unlikely to re-nest after clutch loss	Aquatic insects and invertebrates (esp. amphipods, molluscs)	Pacific coast from Alaska to California

## Materials and methods

### Study area

**Fig. 1:**
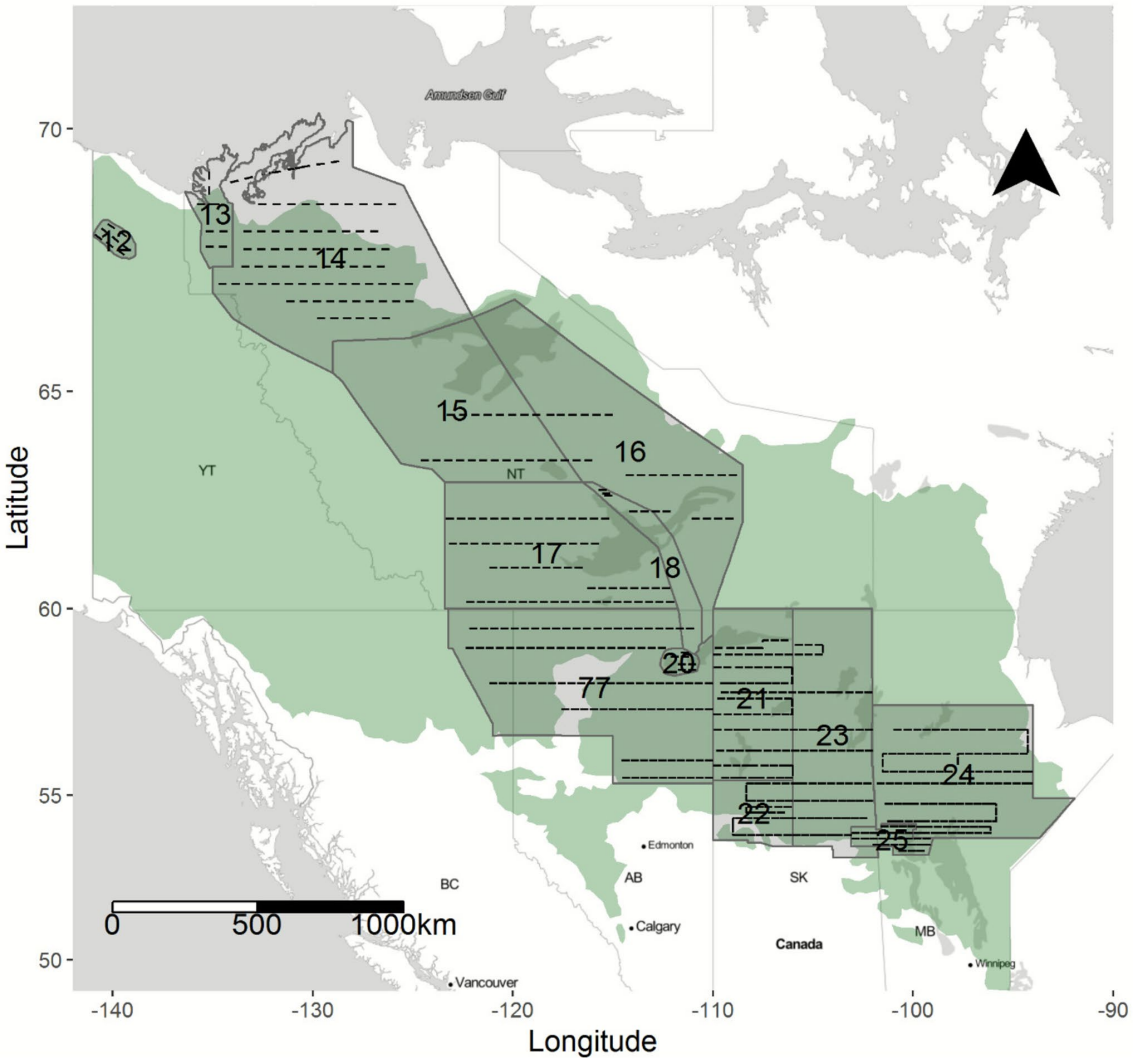
North American Breeding Waterfowl Survey strata (numbered polygons) and transects (dashed lines) overlaid on the Canadian western boreal forest (irregular underlying polygon), which defines the spatial extent of the study area

Our study area covered approximately 617,000 km^2^ of the North American WBF falling within the traditional survey area of the Waterfowl Breeding Population and Habitat Survey (WBPHS; Fig. 1). The region spans a broad latitudinal gradient and includes the mixed deciduous-coniferous forests of the southern boreal plain and Canadian shield regions of Saskatchewan, Manitoba, and Alberta, the predominately coniferous taiga forests of the Northwest Territories, the Yukon Flats wetland complex in the Yukon Territory, and three major river deltas (Saskatchewan, Peace-Athabasca, and Mackenzie). This mosaic of forest and wetland habitats supports a diverse assemblage of boreal-breeding ducks.

### Duck population data

We obtained duck population estimates from the annual WBPHS, conducted by U.S. Fish and Wildlife Service and Canadian Wildlife Service (Figs. 1. and 2; https://iris.fws.gov/APPS/ServCat/Reference/Profile/47314, accessed 31-Dec-2024) for duck species (or species-groups) with breeding ranges that encompassed the entire WBF. Surveys are based on 400-m-wide aerial transects, with breeding pairs counted from fixed-wing aircraft (Smith 1995). The WBF portion of the survey is divided into strata that are broadly delineated by habitat composition and political boundaries (Smith 1995; strata level population data are shown in Online Resource Figure S1). WBPHS dates are adjusted informally each year based on duck migration phenology, spring conditions, and logistic considerations (Smith 1995). Analysis of a shorter period of our study (1982–2013) indicated that stratum average survey dates were not correlated with NDVI spring phenology metrics nor did they trend during the study period (Messmer 2019), implying that survey date variation would not confound estimation of growing season effects. Species included mallard (*Anas platyrhynchos*), American wigeon (*Mareca americana*), green-winged teal (*Anas crecca*), ring-necked duck (*Aythya collaris*), bufflehead (*Bucephala albeola*), generic goldeneye (common [*B. clangula*] and Barrow’s [*B. islandica*]), generic scaup (lesser [*Aythya affinis*] and greater [*A. marila*] scaup), hereafter referred to as ‘scaup’, and generic scoter (surf [*Melanitta perspicillata*], white-winged [*M. deglandi*], and black [*M. americana*] scoters), hereafter referred to as ‘scoter’. “Generic” grouping refers to closely related species which are not readily discernible during aerial surveys. Lesser scaup and common goldeneye are believed to represent the majority of generic scaup and generic goldeneye, respectively, in the study area (Baldassarre 2014).

**Fig. 2:**
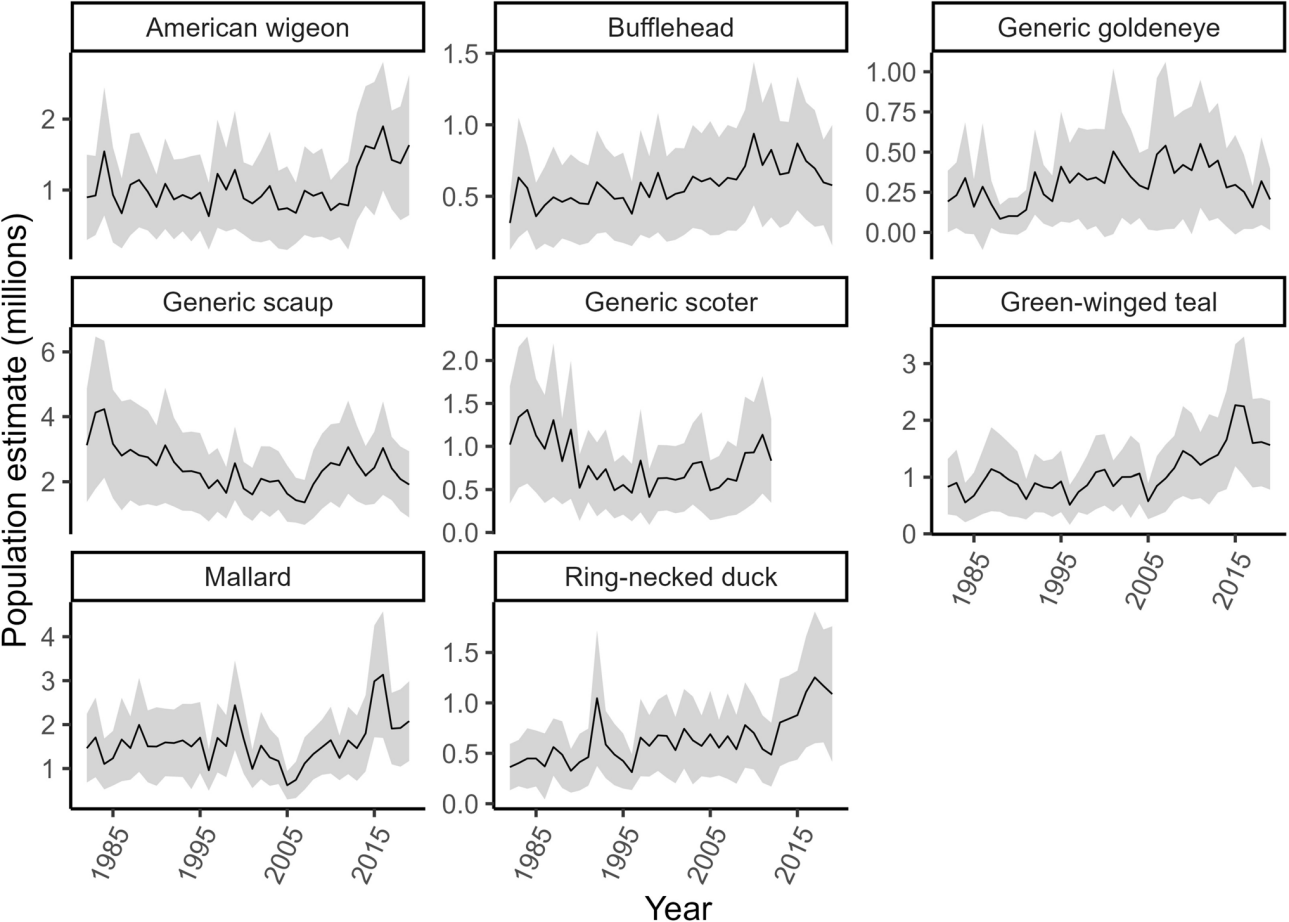
Population estimates (1982–2019) (±95% CI; shaded areas) for all survey strata in the Canadian western boreal forest (see Fig. 1). Estimates are based on data from the U.S. Fish and Wildlife Service Migratory Bird Data Center. Goldeneye, scaup, and scoter estimates include similar species that are not distinguished during aerial surveys (see text for details). Note that breeding population estimates (y-axis scales) vary across species

For each species, strata were excluded from analysis if the species was not recorded in more than five years during the 38-year time series (1982–2019), indicating the area was likely peripheral to its core breeding range and abundance may be more strongly influenced by dispersal from other strata in the WBPHS. For scoter, population estimates were limited to the period 1982–2012, as estimates were discontinued by U.S. Fish and Wildlife Service in 2013.

### NDVI data

We obtained NDVI data from the GIMMS-3G+ (Global Inventory Modeling and Mapping Studies, 3rd generation, version 1.2) dataset (Pinzon et al. 2023; 10.3334/ORNLDAAC/2187, accessed 31-Dec-2024). This dataset includes calibrated NDVI measurements from multiple generations of AVHRR satellite sensors, with a spatial resolution of 8 km and 16-day temporal composites. To characterize growing season dynamics, we used program TIMESAT 3.1.1 (Jönsson and Eklundh 2004) to fit double logistic functions to NDVI time series data for each 64-km^2^ pixel within our study area (Beck et al. 2006; Gao et al. 2008; Barichivich et al. 2013). From these modeled seasonal curves, we extracted the following growing season metrics (interpolated to daily resolution): (i) two variables representing spring phenology: date of start of season (SOS)—calculated as the date when modeled NDVI reaches 25% of that year’s maximum amplitude (Barichivich et al. 2013), and date of peak (DOP) NDVI values—calculated as the date when modeled NDVI reaches that year’s maximum amplitude, (ii) length of the growing season (LOS)—the number of days between SOS and end of season dates (end of season defined as the date when modeled NDVI decreases below 25% of seasonal amplitude), and (iii) growing season photosynthetic productivity (hereafter referred to as productivity)—the area under the seasonal NDVI curve between SOS and end of season dates. SOS, DOP, and LOS are derived relative to seasonal amplitude and are robust to long-term changes in land cover (e.g., wildfires or wetland shifts). In contrast, productivity reflects absolute NDVI values and may be influenced by such changes.

We calculated stratum-level annual averages for each variable and standardized each time series (z-scores: mean = 0, SD = 1) separately by stratum. This ensured that within-stratum effects were isolated in subsequent analyses (van de Pol and Wright 2009). Collinearity among the NDVI-derived variables was assessed using the average Pearson correlation coefficient ($\overline{r }$) across strata.

#### Comparison of NDVI-derived phenology to snow cover duration data

Given previous research examining the influence of spring snow cover duration (SSCD) on duck counts and annual growth rates (Drever et al. 2012; Ross et al. 2015), we compiled SSCD time series in our study area to compare with the NDVI-derived SOS and DOP variables. Because NDVI is sensitive to snow and ice we expected that SOS and DOP would be positively correlated to SSCD. We retrieved weekly snow cover data from the Rutgers University Global Snow Lab (Robinson et al. 1993), with a spatial resolution of 190 km. We calculated SSCD, sensu Drever et al. (2012), as the number of days with snow cover in the second half of the snow season (1 February to 31 July) interpolated to daily resolution. Area-weighted SSCD values were computed for each duck survey stratum. We assessed the relationship between SSCD and both SOS and DOP using Pearson’s correlation coefficient.

### Statistical analysis

To evaluate associations between duck population growth and growing season variables, we implemented species-specific discrete Gompertz population growth models (Forchhammer et al. 1998; Drever et al. 2012; Roy et al. 2016). This approach implicitly models population growth by modeling the current year’s abundance as a function of prior-year abundance and environmental covariates. Given the data structure, we accounted for two key sources of non-independence: (1) observations within strata may be correlated due to stratum-specific intrinsic growth rates, and (2) observations across strata but within years may be correlated due to large-scale synchronizing factors not measured by NDVI covariates, or regional dispersal dynamics (i.e., imperfect breeding site fidelity). To accommodate this structure, we included crossed (i.e., non-nested) random intercepts for both stratum and year (Harrison et al. 2018). We further allowed the residual variance to differ by stratum, acknowledging differences in sampling intensity (number of transects) and ecological variability. Additionally, we included a random slope term for density dependence to allow this parameter to vary by stratum, as supported by previous studies (Sæther et al. 2008; Drever et al. 2012).

Hence, the general model was:

1 $${\mathrm{x}}_{{\mathrm{t,i}}} = \alpha + {\mathrm{stratum}}_{{\mathrm{i}}} + {\mathrm{year}}_{{\mathrm{t}}} + \theta_{{\mathrm{i}}} \times {\mathrm{x}}_{{{\mathrm{t}} - 1, {\mathrm{i}}}} + \beta_{1} \times {\mathrm{NDVI}}\,{\mathrm{covariate}}_{{{\mathrm{t}} - 1,{\mathrm{i}}}} + \varepsilon_{{\mathrm{t,i}}}$$

$${\mathrm{stratum}}_{{\mathrm{i}}} \sim {\mathrm{Normal}}(0,\sigma_{{{\mathrm{stratum}}}}^{2} )$$

$${\mathrm{year}}_{{\mathrm{t}}} \sim {\mathrm{Normal}}(0,\sigma_{{{\mathrm{year}}}}^{2} )$$

$$\theta_{{\mathrm{i}}} \sim {\mathrm{Normal}}(\theta ,\sigma_{\theta }^{2} )$$

$$\varepsilon_{{\mathrm{i,t}}} \sim {\mathrm{MVN}}\left( {0,\,\,\left[ {\begin{array}{*{20}c} {\sigma_{1}^{2} } & \cdots & 0 \\ \vdots & \ddots & \vdots \\ 0 & \cdots & {\sigma_{m}^{2} } \\ \end{array} } \right]} \right)$$

where ${x}_{t,i}$ is the natural log of population density for stratum *i* in year *t*, ${\theta }_{i}$ is the parameter for density dependence, $\alpha$ is the expected grand mean intrinsic growth rate (i.e., growth rate from very low population size), and ${\mathrm{stratum}}_{i}$ and ${\mathrm{year}}_{t}$ are year and site random intercept terms (Kéry and Schaub 2012). The term ${e}_{t,i}$ represents random variation in observed population growth rate not explained by covariates or caused by observation error and we assumed a multivariate normal (MVN) distribution, independent for each stratum *i* = 1,…,*m*. NDVI covariate_t-1,i_ is the NDVI-derived index (i.e., SOS, DOP, LOS, or productivity), and βs are estimated coefficients for these terms.

We estimated model parameters using restricted maximum likelihood (REML) as implemented in the *nlme* package (version 3.1-166; Pinheiro et al. 2018) for R (version 4.4.2; R Core Team 2024). Model assumptions, particularly regarding the homoscedasticity of residuals, were assessed following procedures described in Zuur et al. (2009) for each species. We focused ecological interpretations solely on the fixed effect estimates relevant to our hypotheses. Estimates of random effects and the density dependence parameter were not interpreted due to concerns about bias arising from observation error (Dennis and Taper 1994) and the inherent confounding of population growth rate and density dependence in such models (Roy et al. 2016). Nonetheless, fixed effect estimates for non-autocorrelated covariates are generally considered robust and unbiased in this modeling framework (Lindén and Knape 2009).

#### Modeling strategy

We first evaluated collinearity among the growing season variables, as multicollinearity can complicate the estimation and interpretation of parameters in regression analysis, particularly when the partial effect of a given variable is of interest (Dormann et al. 2013; Cade 2015). Next, for each species group, we fit Gompertz models separately for each of the lag-1 growing season covariates, both as a main effect and in interaction with latitude. Interactions with latitude were used to account for the possibility that effects would be more pronounced at higher latitudes, where growing seasons are typically shorter. If the interaction effect was supported by a *p*-value < 0.05 we interpreted covariate effects relative to latitude. We applied the Benjamini–Hochberg (BH) false discovery rate (FDR) adjustment to effect estimates’ *p*-values, recognizing that FDR control is increasingly recommended in ecological analysis of multiple hypotheses (Benjamini and Hochberg 1995; Waite and Campbell 2006). Adjusted *p*-values (i.e., BH *q*-values) were calculated across the full set of 64 tests (8 species × 4 NDVI main effect terms × 4 latitude interaction terms). We considered effects with adjusted BH *q*-value < 0.05 statistically robust. For visualization, we present effect estimates with their unadjusted 95% confidence intervals, as we applied the FDR correction to *p*-values rather than to the intervals themselves. This approach allows direct comparison of effect magnitudes and uncertainty with conventional intervals while maintaining appropriate control of FDR across tests by flagging results that exceed the BH q-value threshold. For LOS, we included SOS in the model to account for the logical relationship that LOS = End of Season − SOS. We standardized the latitude variable as latitude_i_ − min(latitude_i_), such that the growing season variable’s reported coefficient was the estimated effect at the lowest latitude stratum and a positive sign for the interaction indicates that the effect increases with latitude.

## Results

There was no consistent temporal trend in date of start of season (SOS) across the study period (Online Resource Figure S2). However, the date of peak NDVI (DOP) advanced (i.e., became earlier) over time, particularly in the northernmost survey strata (Online Resource Figure S3). Length of the growing season (LOS) generally declined toward the end of the study period, with this trend most pronounced at higher latitudes (Online Resource Figure S4). Productivity followed a curvilinear temporal pattern across many strata, with peak values typically occurring in the middle of the time series, especially at northern latitudes (Online Resource Figure S5). The spatial variability in mean seasonal metrics across survey strata was as follows: SOS ranged by 30.2 days on average (SD = 4.7), DOP by 18.9 days (SD = 2.7), and LOS by 61.3 days (SD = 10.0).

Correlations among growing season variables revealed expected associations. SOS and DOP were moderately correlated ($\overline{r }$ = 0.50); earlier SOS was generally associated with earlier DOP. SOS exhibited negative correlations with LOS ($\overline{r }$ = −0.68) and productivity ($\overline{r }$ = −0.52), indicating that growing seasons with earlier springs tended to be longer and more productive. LOS and productivity were strongly positively correlated ($\overline{r }$ = 0.75), while productivity was uncorrelated with DOP ($\overline{r }$ = −0.08). LOS and DOP were also uncorrelated ($\overline{r }$ = 0.03). The spring snow cover duration (SSCD) index used in earlier studies (e.g., Drever et al. 2012; Ross et al. 2015) showed moderate correlation with SOS ($\overline{r }$= 0.63).

**Fig. 3:**
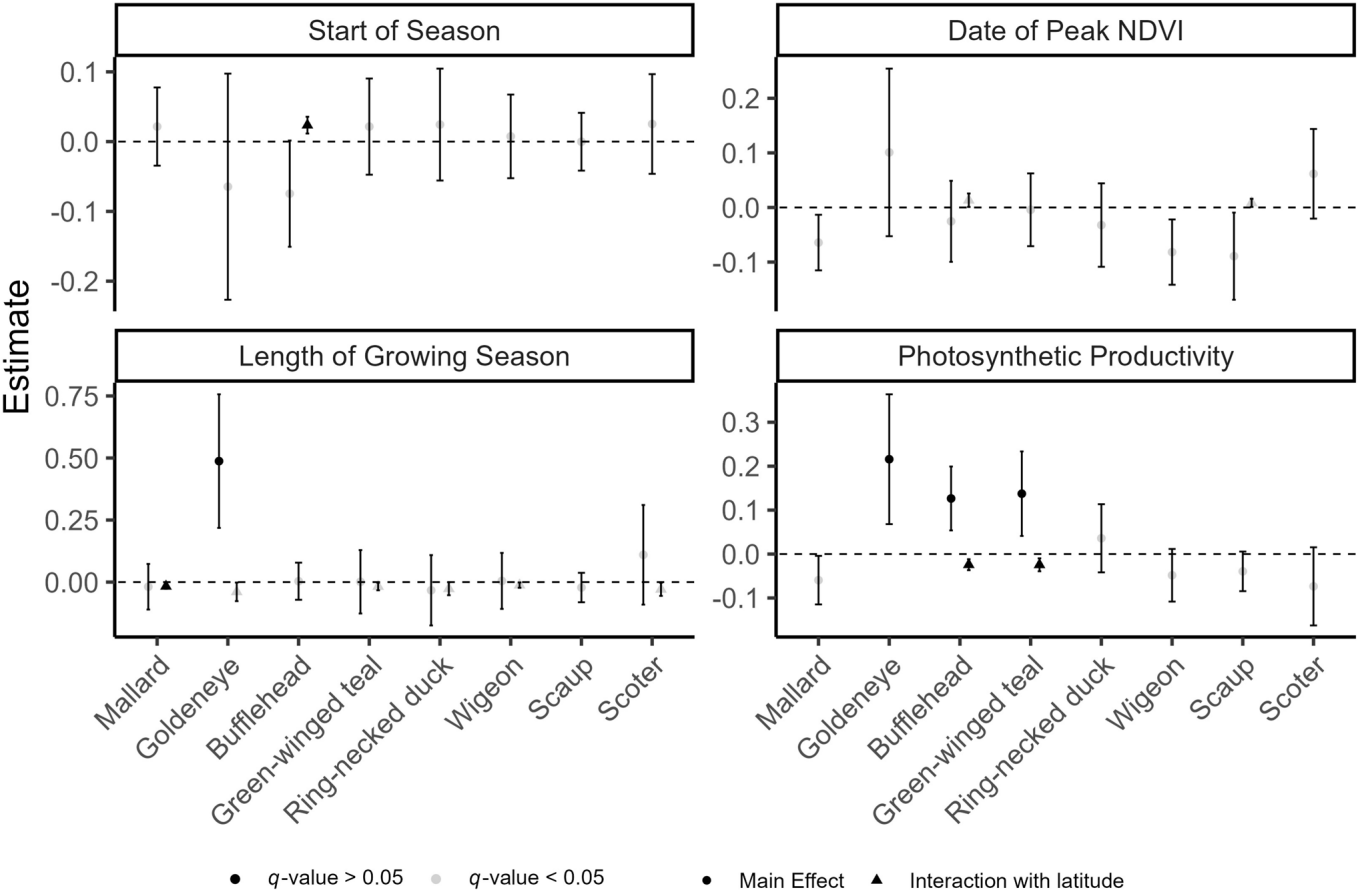
Standardized regression coefficient estimates (±95% CI) for growing season covariates affecting annual population growth. Covariates supported by Benjamini–Hochberg false discovery rate adjustment *q*-value < 0.05 are shown *black*, whereas unsupported covariates are grey. Covariates were standardized (mean = 0, SD = 1), allowing interpretation as the percent change in population growth per 1 SD change in the covariate. Interaction terms with latitude (triangle symbol) were included when supported by unadjusted *p*-value < 0.05. Latitude was standardized as ‘latitude − min(latitude)’ thus, main effects represent estimates at the southernmost latitude of the study area, and interaction terms reflect the additional change in effect per 1° increase in latitude

Among the 8 species or species groups, we detected statistically significant effects of the four NDVI-derived growing season variables—SOS, DOP, LOS, and productivity—on the annual population growth rates of 1, 0, 2, and 3 species or species groups, respectively, at the BH *q*-value threshold ≤0.05 (Fig. 3). All effects varied with latitude, except the goldeneye-productivity relationship, indicating differential responses across the boreal breeding range.

We found mixed support for the hypothesis that species’ responses to growing season phenology align consistently with their average timing of breeding (Fig. 3; Table 2). Specifically, we found a general lack of species responses to SOS and DOP across the gradient of species average nesting dates, with only one statistically supported relationship with BH *q*-value < 0.05: bufflehead showed a positive association between SOS and growth rate at high latitudes—the opposite of our prediction for this early-breeding species. While absence of an effect matched predictions for early nesting species, the late-nesting species groups, scaup and scoter, did not have the predicted negative response.

**Table 2:** Summary of prediction outcomes for growing season variable impacts on population growth rates

Species	Nesting phenology	Start of Season	Date of peak	Length of Season	Productivity
Mallard	Early	Yes^a^	Yes^a^	No (opposite at high latitudes)	No^a^
Goldeneye	Early	Yes^a^	Yes^a^	Yes (at low latitudes)	Yes
Bufflehead	Early	No (opposite at high latitudes)	Yes^a^	No^a^	Yes (only at low latitudes)
Green-winged teal	Mid	Yes^a^	Yes^a^	No^a^	Yes (only at low latitudes)
Ring-necked duck	Mid	Yes^a^	Yes^a^	No^a^	No^a^
American Wigeon	Mid	Yes^a^	Yes^a^	No^a^	No^a^
Scaup	Late	No^a^	No^a^	No^a^	No^a^
Scoter	Late	No^a^	No^a^	No^a^	No^a^

For seasonal phenology variables (start of season and date of peak), early-nesting species were predicted to show no response or a negative response to later phenology, while late-breeding species were predicted to respond positively. For length of season and productivity, all species were predicted to respond positively, regardless of nesting phenology. “Opposite” (in parentheses) indicates an effect was detected contrary to the prediction. Conclusions were based on Benjamini–Hochberg multiple-hypothesis testing adjustment *q*-value < 0.05

^a^No effect detected

Effects of LOS were absent in all species except early-breeding mallard and goldeneye (Fig. 3). In mallard the effect was neutral at lower latitudes but became more negative at higher latitudes—opposite to our hypothesis that longer growing seasons would increase population growth. There was a similar pattern in green-winged teal, ring-necked duck, American wigeon, and generic scoter (Fig. 3), although these were not supported after multiple hypothesis testing adjustment. Only in generic goldeneye did LOS show the predicted positive effect, and this relationship decreased with latitude (Table 2; Fig. 3). In contrast, productivity had the hypothesized positive effect on growth rates in three species: generic goldeneye, bufflehead, and green-winged teal (the latter two only at low latitudes; Fig. 3, Table 2). For the remaining five species, productivity had no detectable effect.

## Discussion

We analyzed a 38-year dataset (1982–2019) of duck breeding population growth rates to assess whether interannual variation in NDVI-inferred growing season characteristics—phenology, length, and productivity—affected duck dynamics in the WBF. While the impacts of shifting phenology have been documented in various avian taxa, our study is among the first to explicitly evaluate the effects of growing season length and cumulative productivity on secondary consumer species.

Contrary to expectations given documented climate warming across much of North America (IPCC 2007), and advancing start of growing season across much of the northern hemisphere, we found no consistent long-term trends toward earlier start of season (SOS), longer length of season (LOS), or increased NDVI productivity. However, there is substantial spatial heterogeneity in these trends globally (Garonna et al. 2016; Liu et al. 2023), and our findings align with those of Barichivich et al. (2013), who also reported no significant shifts in thermal or photosynthetic growing seasons across a broader swath of northern North America during a similar timeframe (1982–2011).

We found mixed support for the hypothesis that variation in growing season phenology affects duck population growth rates through timing-of-breeding mechanisms. Consistent with the evidence that early- and mid-season nesting species generally track environmental phenology cues (Oja and Pöysä 2007; Dessborn et al. 2009; Gurney et al. 2011; Messmer et al. 2021), we detected no clear DOP effects and a single SOS effect amongst these species, which was positive. However, contrary to expectations from Drever et al. (2012), late-nesting species which generally exhibit less nesting date flexibility (Raquel et al. 2016; Messmer et al. 2021) were not negatively impacted by earlier SOS. This result corroborates Ross et al. (2015) who did not detect a spring snow phenology impact in scaup over a more extensive study area and time period. Taken together, the SOS and DOP findings do not support a clear timing-of-breeding mediated response consistent with the phenological mismatch hypothesis. Rather, it appears that the species studied generally exhibit sufficient plasticity in life-history traits to accommodate the observed interannual variation in spring phenology (Drever and Clark 2007; Sjöberg et al. 2011; Arzel et al. 2014; Clark et al. 2014), or that compensatory mechanisms elsewhere in the life cycle buffer potential mismatches (Reed et al. 2013).

The absence of consistent negative effects of phenological variation on scaup and scoter is particularly notable. These species groups experienced substantial declines across the WBF from the mid-1980s to mid-2000s (Afton and Anderson 2001; Ross et al. 2012), and spring phenology-driven trophic mismatch has been proposed as a contributing factor (Drever et al. 2012). Although there was weak support for a negative DOP effect on scaup growth at low latitudes if we had not adjusted our testing criteria for multiple hypothesis tests, this pattern could not explain continental-scale declines, as the southern strata are used by a small portion of the continental breeding population and DOP patterns do not align temporally with population trends. Taken together with the results of Ross et al. (2015), we suggest phenological mismatch caused by advancing spring phenology was not the cause of scaup and scoter declines observed in the study area. Evidence that low breeding productivity drove negative growth rates during lesser scaup population declines (Hobson et al. 2009; Koons et al. 2017) may instead reflect other factors, such as predation rates (Brook et al. 2005) or food web changes unrelated to growing season variation (Corcoran et al. 2009).

We hypothesized that longer growing seasons would positively influence population growth by extending opportunities for renesting and prolonging offspring-rearing periods (Halupka and Halupka 2017). Contrary to expectations, we detected no response for six species and a negative interactive effect of growing season length with latitude for mallard. Although not supported after multiple-hypothesis testing adjustment, a similar pattern to mallard was also evident in wigeon, green-winged teal, ring-necked duck, and generic scoter. Counter-intuitively, the negative effect was strongest at higher latitudes for each species, where the length of the growing season is already most limited. Thus, outside of generic goldeneye in high latitude strata, there was no evidence that longer growing seasons corresponded with improved reproductive outcomes, as reported in other species and regions (Halupka and Halupka 2017; Halupka et al. 2021). It is possible that the additional time afforded by a longer season is insufficient for successful renesting, or for ducklings from later broods to fledge and initiate migration in good body condition. If extended seasons increase renesting attempts without a corresponding increase in offspring recruitment, they may simply expose females to higher cumulative nesting mortality risk (Devries et al. 2003; Brasher et al. 2006) and poorer body condition at the onset of fall migration. This may help explain the attenuation of negative effects for mallard at lower latitudes, where renesting attempts are more likely to result in successful fledging and migration. Nonetheless, other ecological processes may explain this pattern. Of these possibilities, retention-dispersal dynamics between prairie and boreal duck populations during prairie flood and drought periods could impact boreal duck dynamics. However, during our study period annual estimates of prairie ponds from the WBPHS were not correlated with the mean NDVI covariates across our study area (all Pearson *r* < 0.17 and corresponding *p*-values > 0.05), indicating this potential dynamic did not confound our analysis.

We also expected that higher cumulative growing season productivity would increase population growth through bottom-up trophic effects and by signaling favorable hydrological conditions. While goldeneye and low-latitude bufflehead and green-winged teal populations responded positively, no effects were detected for the other five species. Although primary productivity has been linked to species richness and spatial distributions (Rosenzweig and Abramsky 1993; Mittelbach et al. 2001; Ding et al. 2006; Pettorelli et al. 2009), our study was among the first to assess population-level impacts of annual productivity fluctuations on secondary consumer species. Our results could also imply that terrestrial NDVI-based productivity is not a sufficiently strong proxy for aquatic productivity relevant to breeding waterfowl. This conclusion is supported by studies showing no effect of wildfire—another driver of terrestrial productivity—on duck breeding distribution in some northern systems (Haszard and Clark 2007; Lewis et al. 2016). Nonetheless, NDVI productivity indices still serve as indicators of drought absence (Peters et al. 2002; Maselli et al. 2003), which has known benefits for breeding ducks (Dzus and Clark 1998), possibly suggesting boreal wetlands and duck populations may not be as drought-affected as those in prairie and parkland regions.

Overall, our results suggest that indirect biotic mechanisms, rather than direct abiotic or primary productivity influences, could mediate the effects of climate on populations at higher trophic levels (Shurin et al. 2012; Ockendon et al. 2014), such as those occupied by ducks. This contrasts with more herbivorous species, such as arctic-nesting geese, for which growing season conditions appear to exert more direct effects (Brook et al. 2015; Ross et al. 2017, 2018). For ducks, processes operating over longer temporal scales may overshadow more immediate, short-term climatic influences. These influences may include predator population dynamics (Brook et al. 2005; Ross et al. 2015; Dyson et al. 2020), wetland inundation dynamics (Mialon et al. 2005; Huang et al. 2022; Merchant et al. 2024), and multi-year shifts in prey composition and abundance (Gerten and Adrian 2002; Corcoran et al. 2009; Gurney et al. 2017; Bush et al. 2020). Future research into boreal duck population dynamics should attempt to account for the role of biotic interactions and multi-scale processes, while also incorporating putative effects of seasonal climate metrics. Integrating ecological complexity and long-term feedbacks may be essential to understanding past duck dynamics and improving predictions under ongoing environmental change.

## Supplementary Information

Below is the link to the electronic supplementary material.

Supplementary file1 (PDF 1791 KB)

## Data Availability

The datasets used during the current study are publicly available from the official sources cited in text.

## Code availability

Analysis code used during the current study is available upon request from the corresponding author.
