# Supplementary material for: Divergent demographic responses of boreal-breeding ducks to growing season variability
Source: Oecologia. 2026 Feb 4;208(2):28. doi: 10.1007/s00442-026-05865-x (PMC12872709; doi:10.1007/s00442-026-05865-x)

Electronic Supplemental Material for:

Title: Divergent demographic responses of boreal-breeding ducks to growing season variability

Authors:

David J. Messmer[1], Stuart Slattery[2], Mark C. Drever[3*], Chris Derksen[4] and Robert G. Clark[1,5].

[1] Department of Biology, 112 Science Place, University of Saskatchewan, Saskatoon, Saskatchewan S7N 2E5, Canada.

[2] Ducks Unlimited Canada, Institute for Wetland and Waterfowl Research, PO Box 1160, Stonewall, MB, R0C 2Z0 Canada

[3] Pacific Wildlife Research Centre, Environment & Climate Change Canada, 5421 Robertson Road, Delta, British Columbia, V4K 3N2 Canada

[4] Climate Processes Section, Environment & Climate Change Canada, 4905 Dufferin Street, Toronto, Ontario, M3H 5T4 Canada

[5] Prairie and Northern Wildlife Research Centre, Environment & Climate Change Canada, Saskatoon, Saskatchewan, S7N 0X4 Canada

*Correspondence to: mark.drever@ec.gc.ca, 1-250-327-4101

Figure S1) Population estimates (1982–2019) (± 95% CI; shaded areas) for survey strata in the Canadian western boreal forest (see Figure 1). Estimates are based on data from the U.S. Fish and Wildlife Service Migratory Bird Data Center. Goldeneye, scaup, and scoter estimates include similar species that are not distinguished during aerial surveys (see text for details). Note that breeding population estimates (y-axis scales) vary across species. Each species is a separate multi-panel plot and panel labels correspond to stratum names shown in Figure 1 and are arranged in order of decreasing mean latitude.

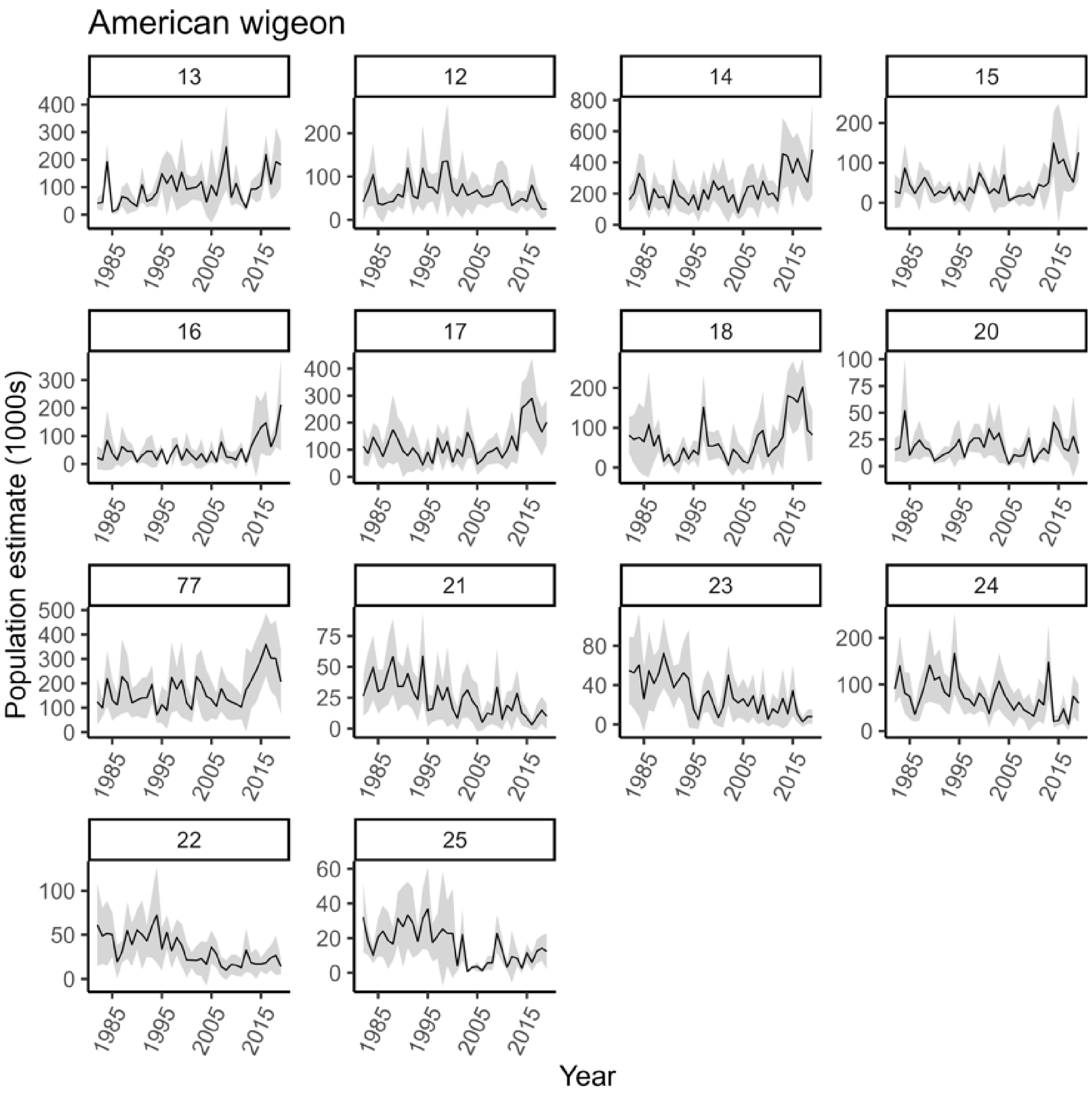
American wigeon
13
12
14
15
16
17
18
20
77
21
23
24
22
25
Population estimate (1000s)
Year
1985
1995
2005
2015

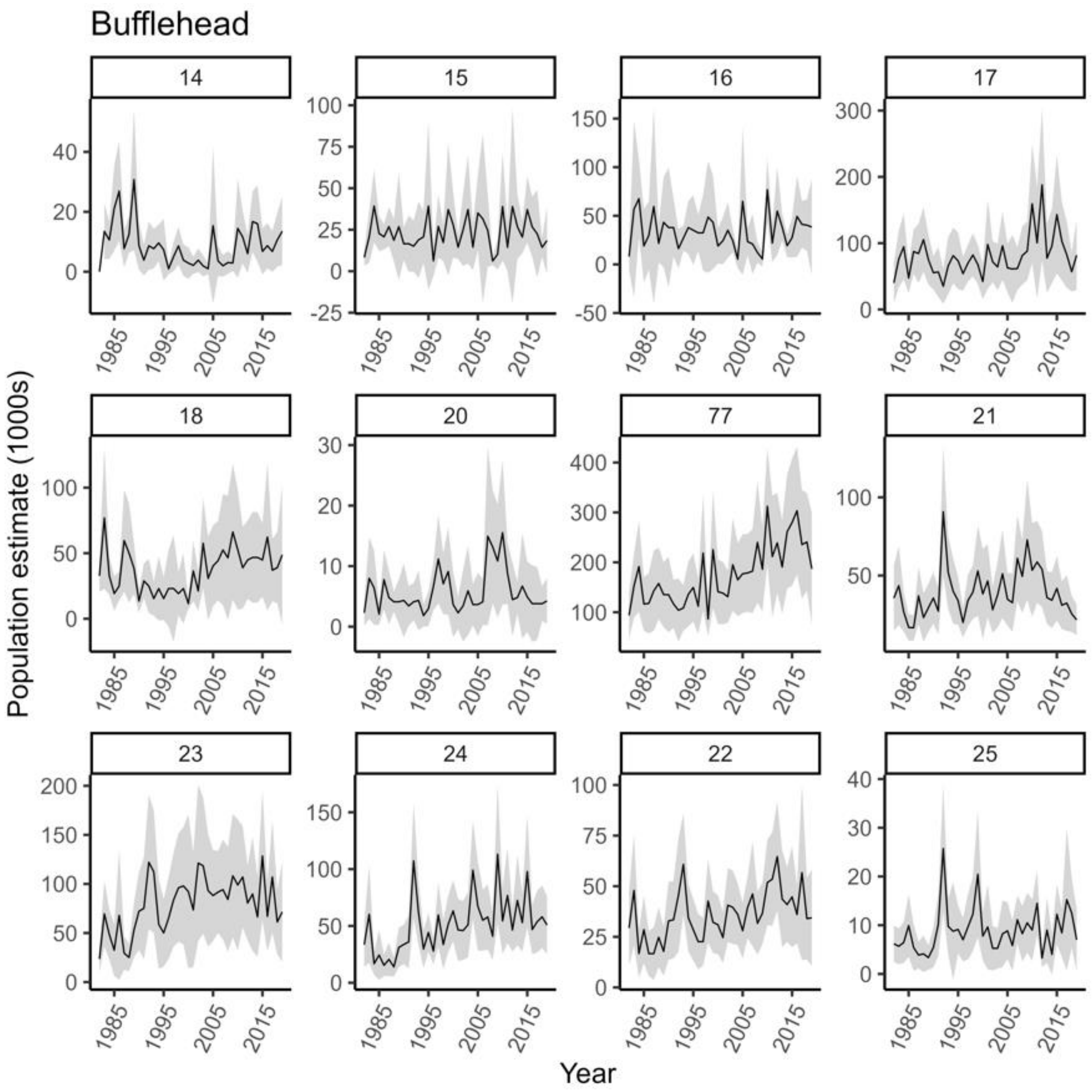

Bufflehead
14
15
16
17
18
20
77
21
23
24
22
25
Population estimate (1000s)
Year
1985
1995
2005
2015

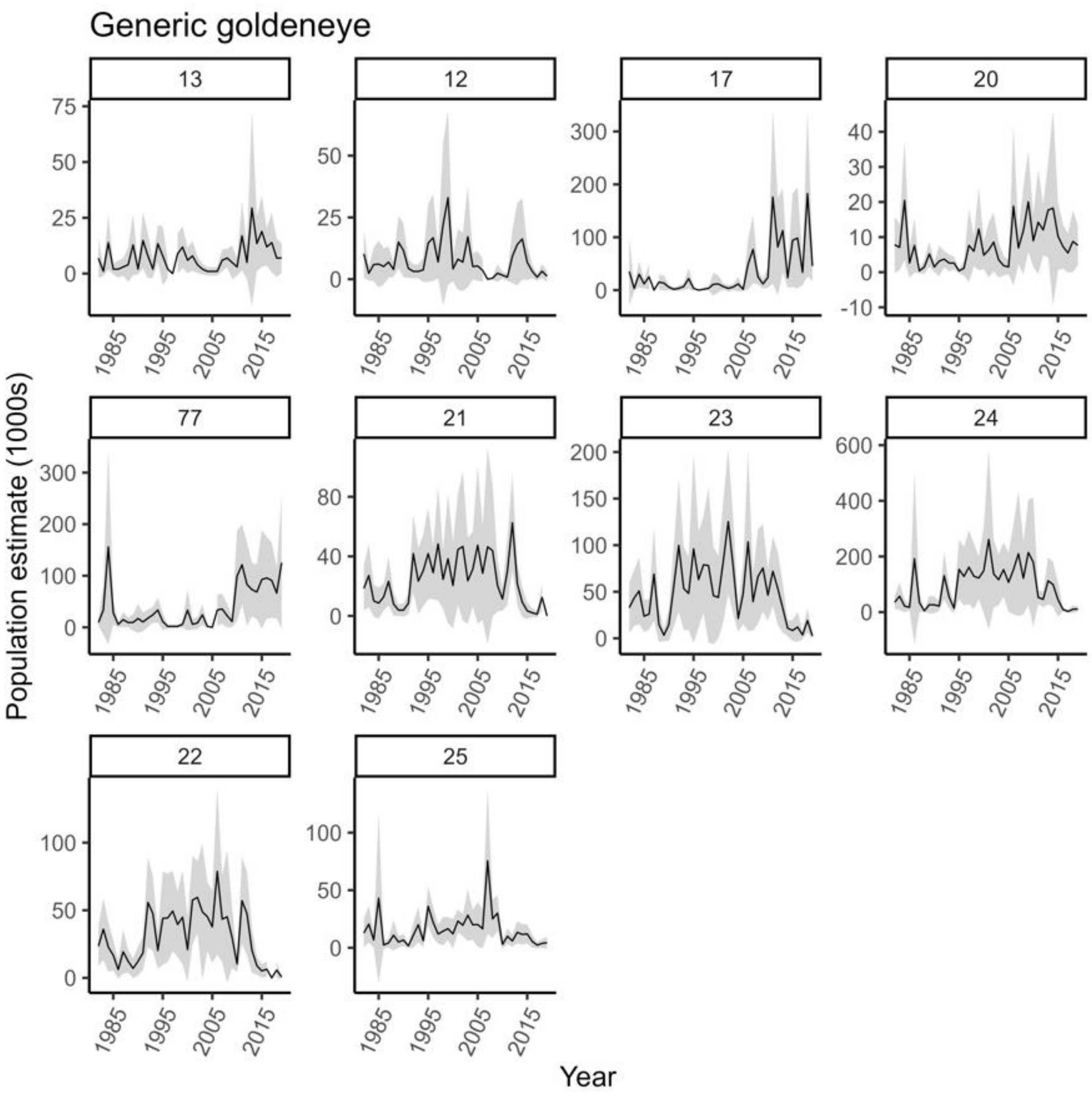

Generic goldeneye
13
12
17
20
77
21
23
24
22
25
Population estimate (1000s)
Year
1985
1995
2005
2015

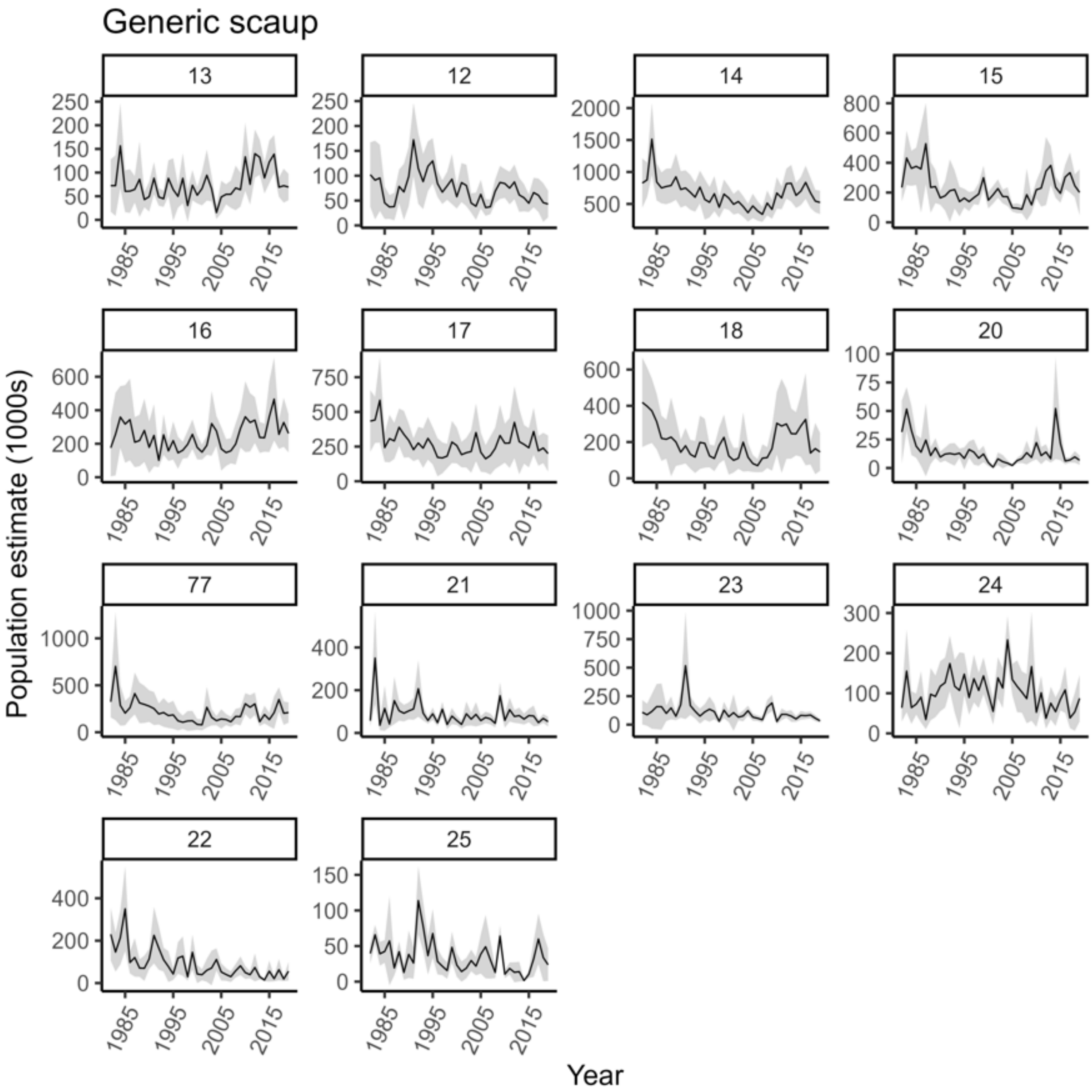

Generic scaup
13
12
14
15
16
17
18
20
77
21
23
24
22
25
Population estimate (1000s)
Year

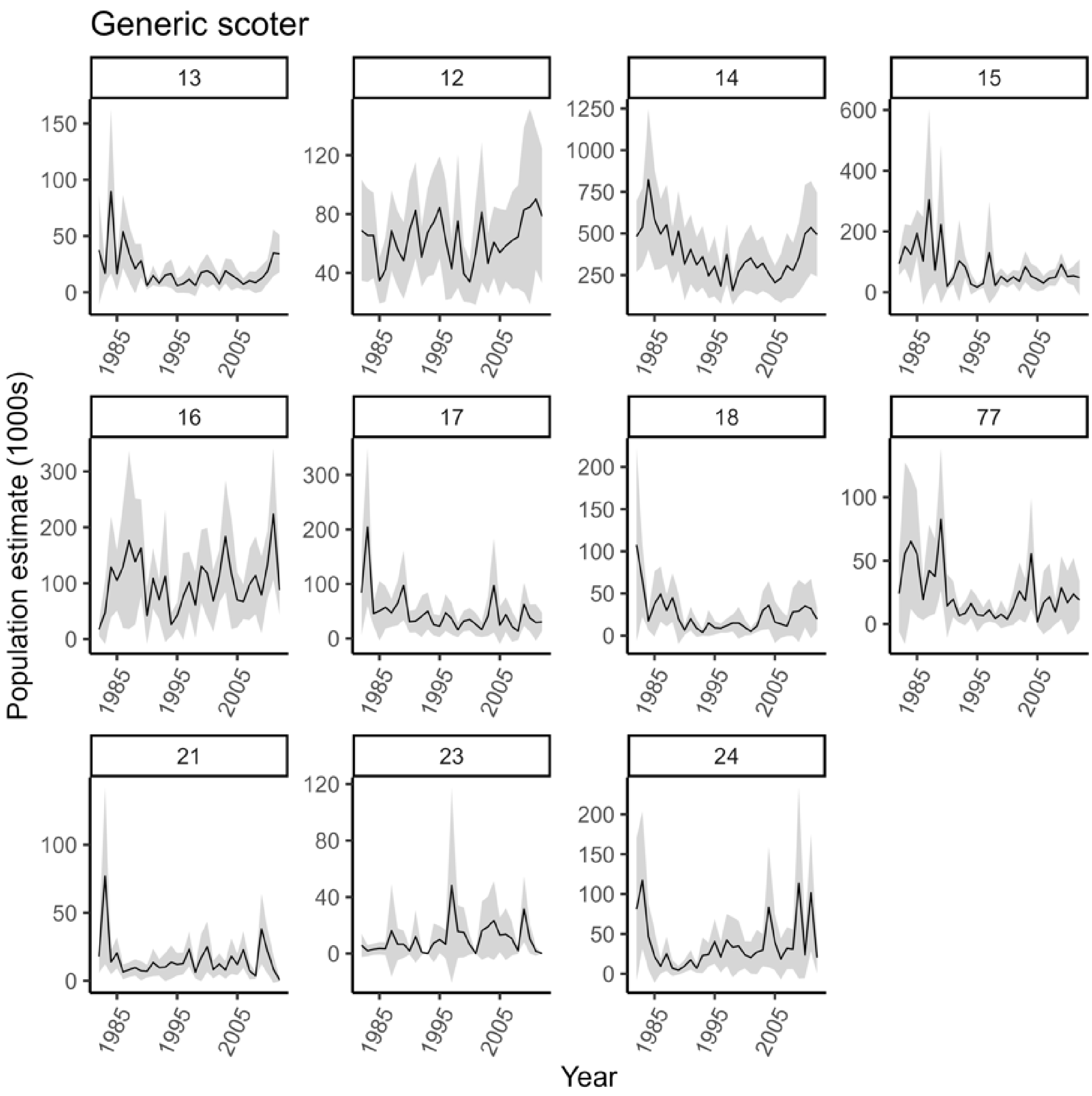
Generic scoter
13
12
14
15
16
17
18
77
21
23
24
Population estimate (1000s)
Year
1985
1995
2005

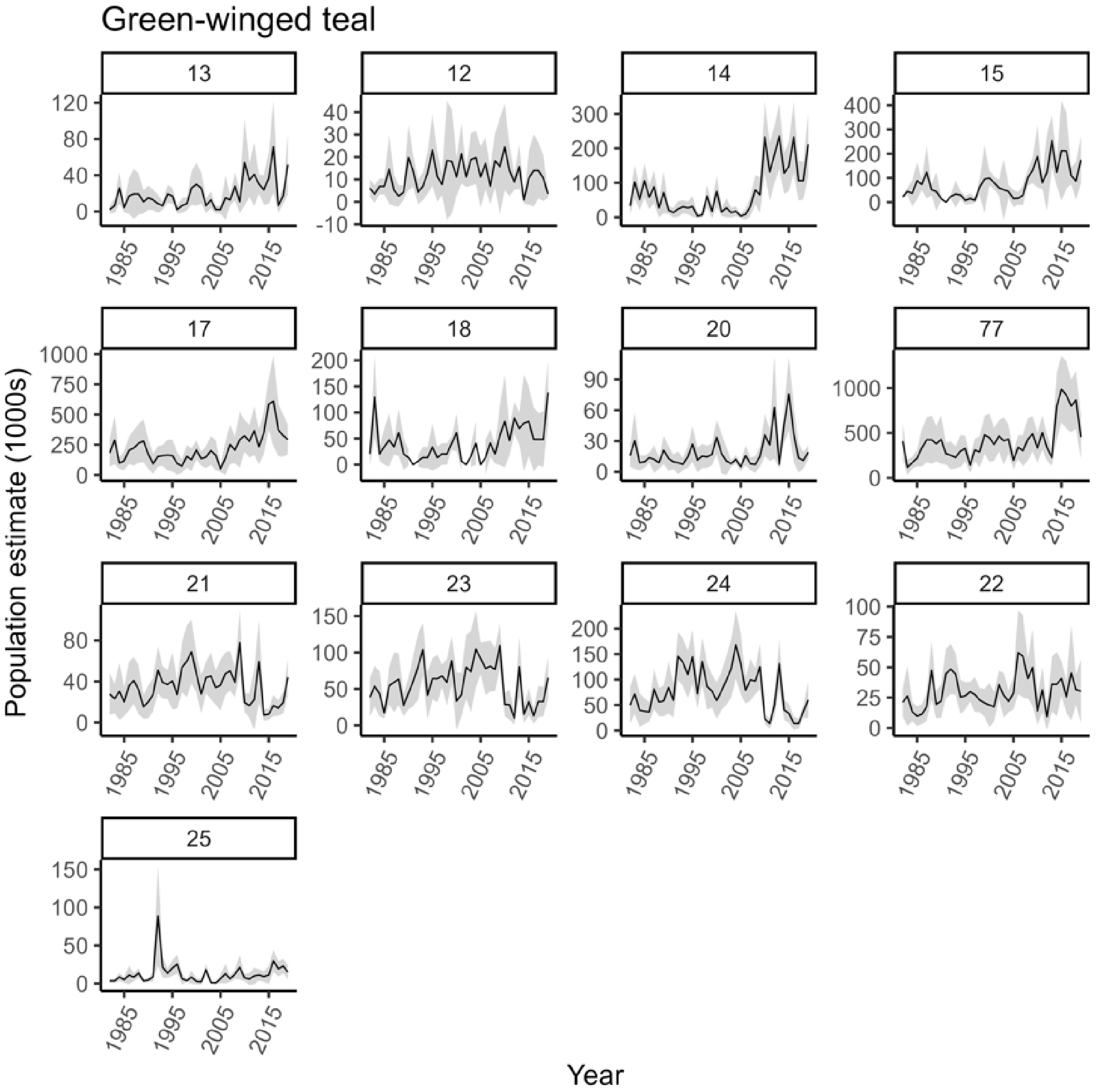

Green-winged teal
13
12
14
15
17
18
20
77
21
23
24
22
25
Population estimate (1000s)
Year

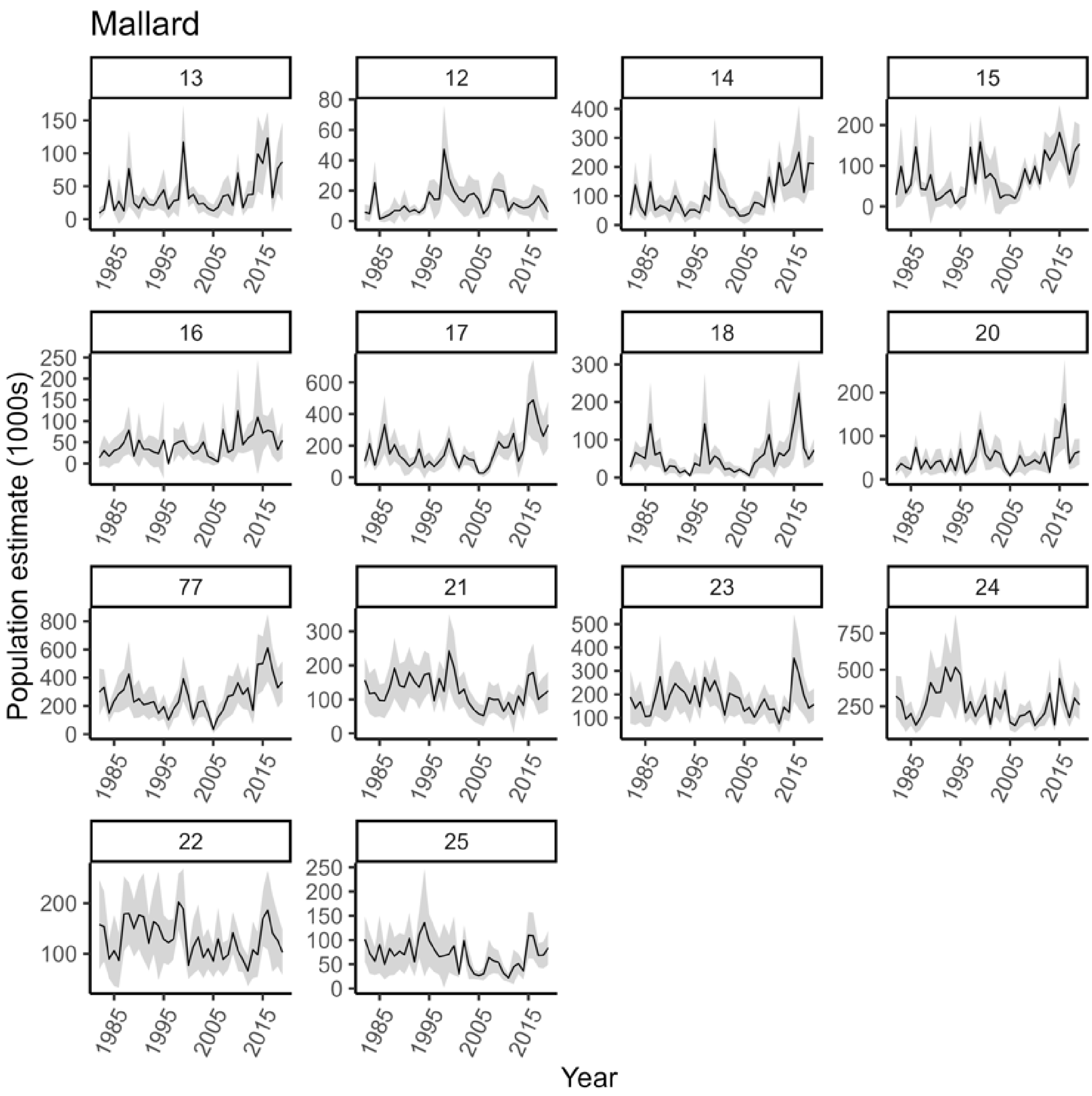

Mallard
13
12
14
15
16
17
18
20
77
21
23
24
22
25
Population estimate (1000s)
Year

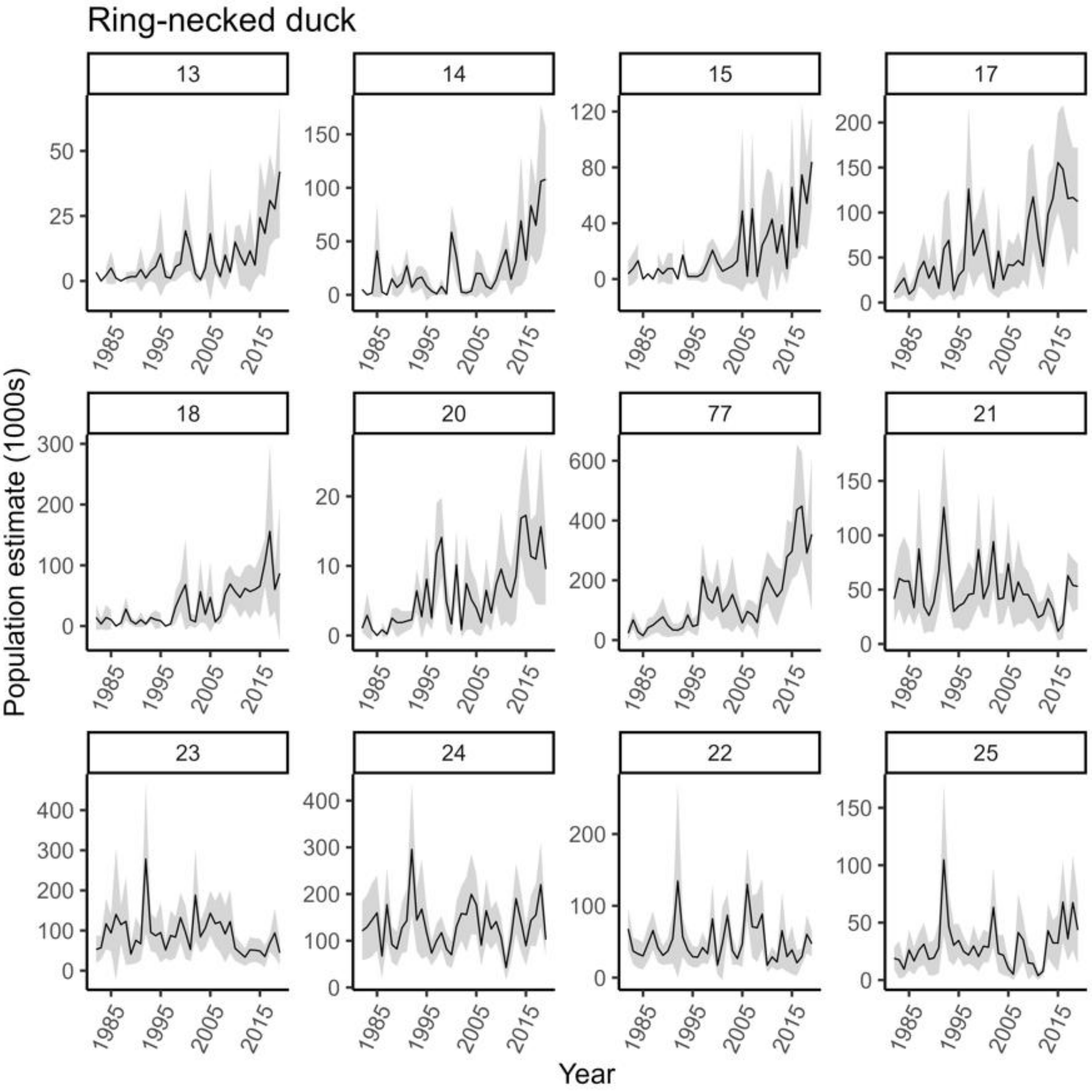

Ring-necked duck
13
14
15
17
18
20
77
21
23
24
22
25
Population estimate (1000s)
Year

Figure S2) Time series of start-of-season day of year (1982–2019) inferred from the normalized difference vegetation index (NDVI), using the 25% seasonal amplitude threshold, for each duck survey stratum in the western boreal forest of Canada. Panel labels correspond to stratum names shown in Figure 1 and are arranged in order of decreasing mean latitude.

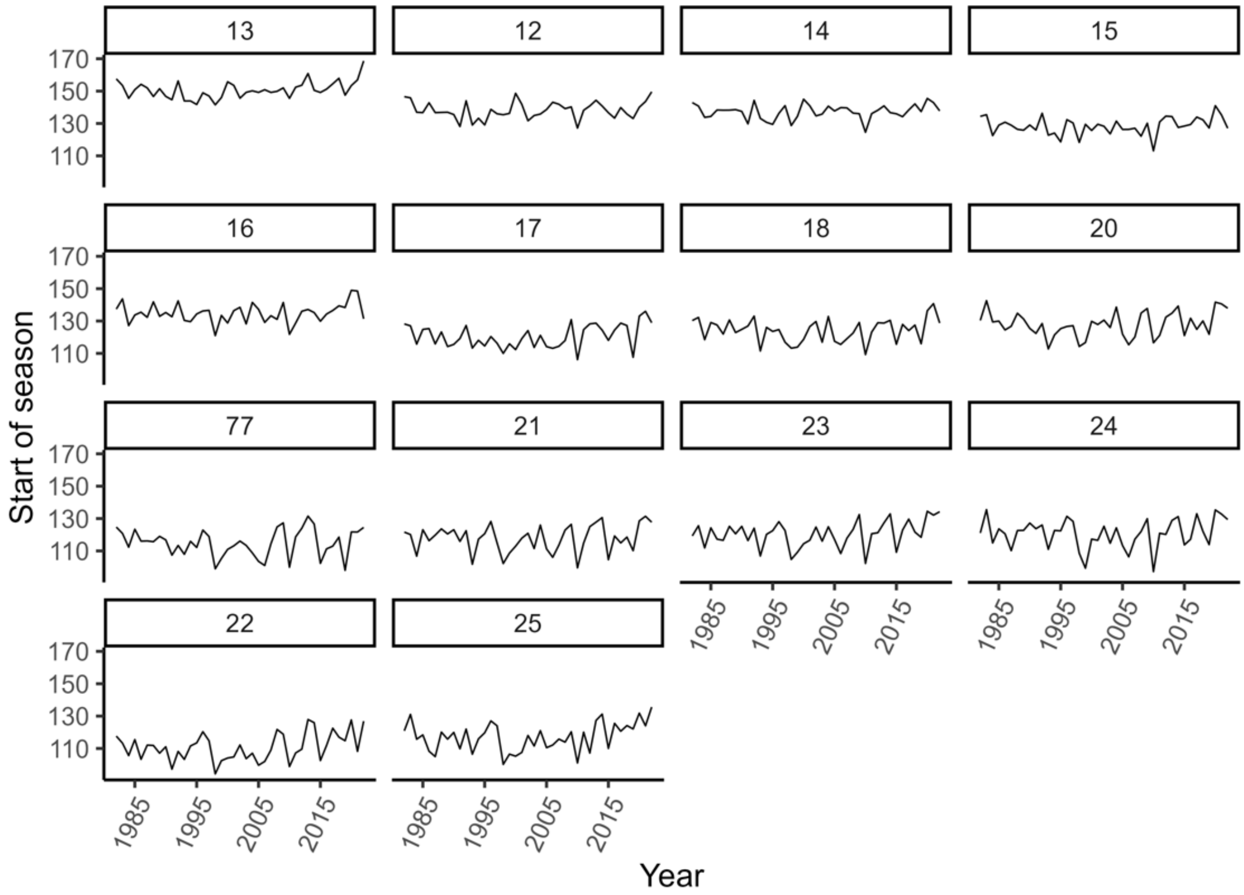

Figure S3) Time series of day of year of peak modeled normalized difference vegetation index (NDVI) for each growing season (1982–2019), for each duck survey stratum in the western boreal forest of Canada. Panel labels correspond to stratum names shown in Figure 1 and are arranged in order of decreasing mean latitude.

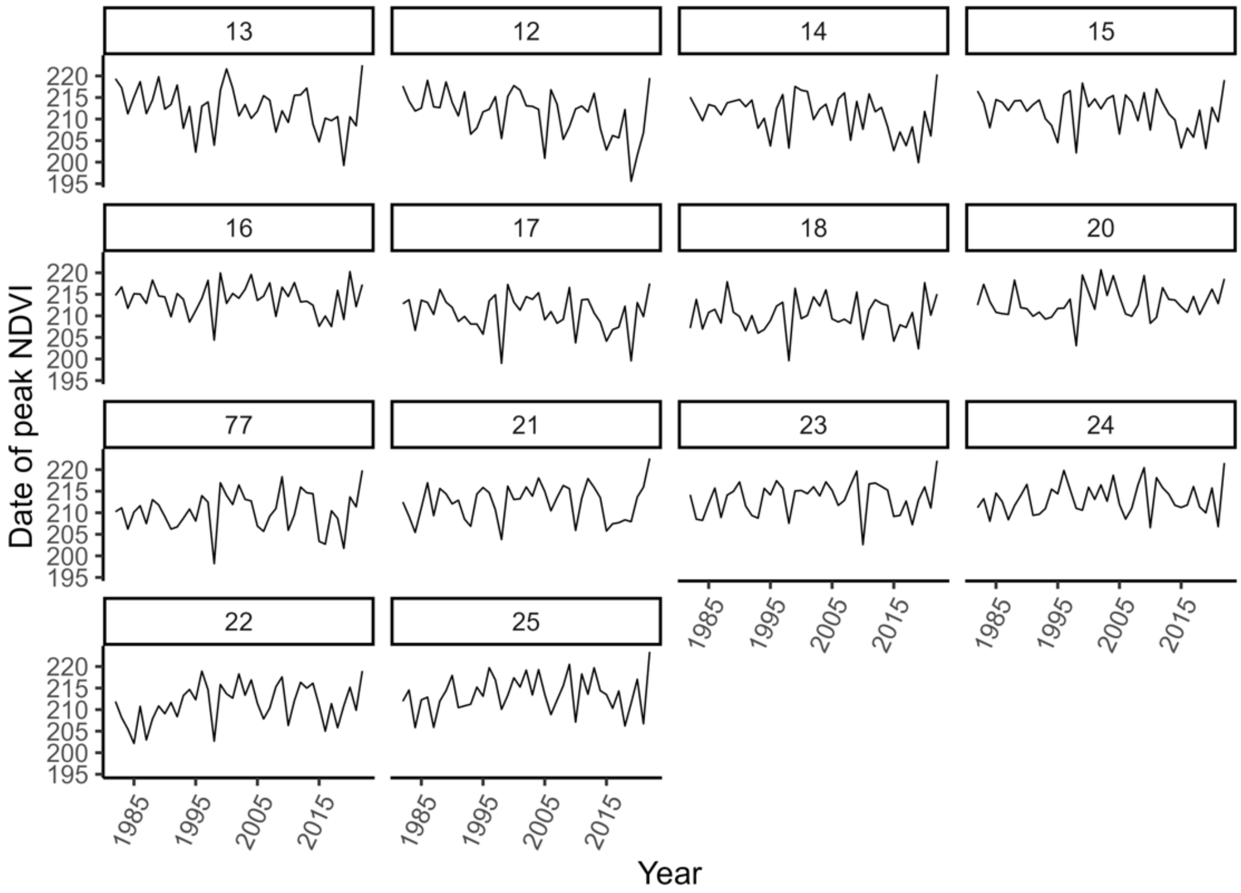

Figure S4) Time series of growing season length (in days) from 1982 to 2019, inferred from the normalized difference vegetation index (NDVI) for each duck survey stratum in the western boreal forest of Canada. Season length was calculated as the difference between the day of year corresponding to the upper and lower 25% of the seasonal amplitude of the modeled NDVI curve. Panel labels correspond to stratum names shown in Figure 1 and are arranged in order of decreasing mean latitude.

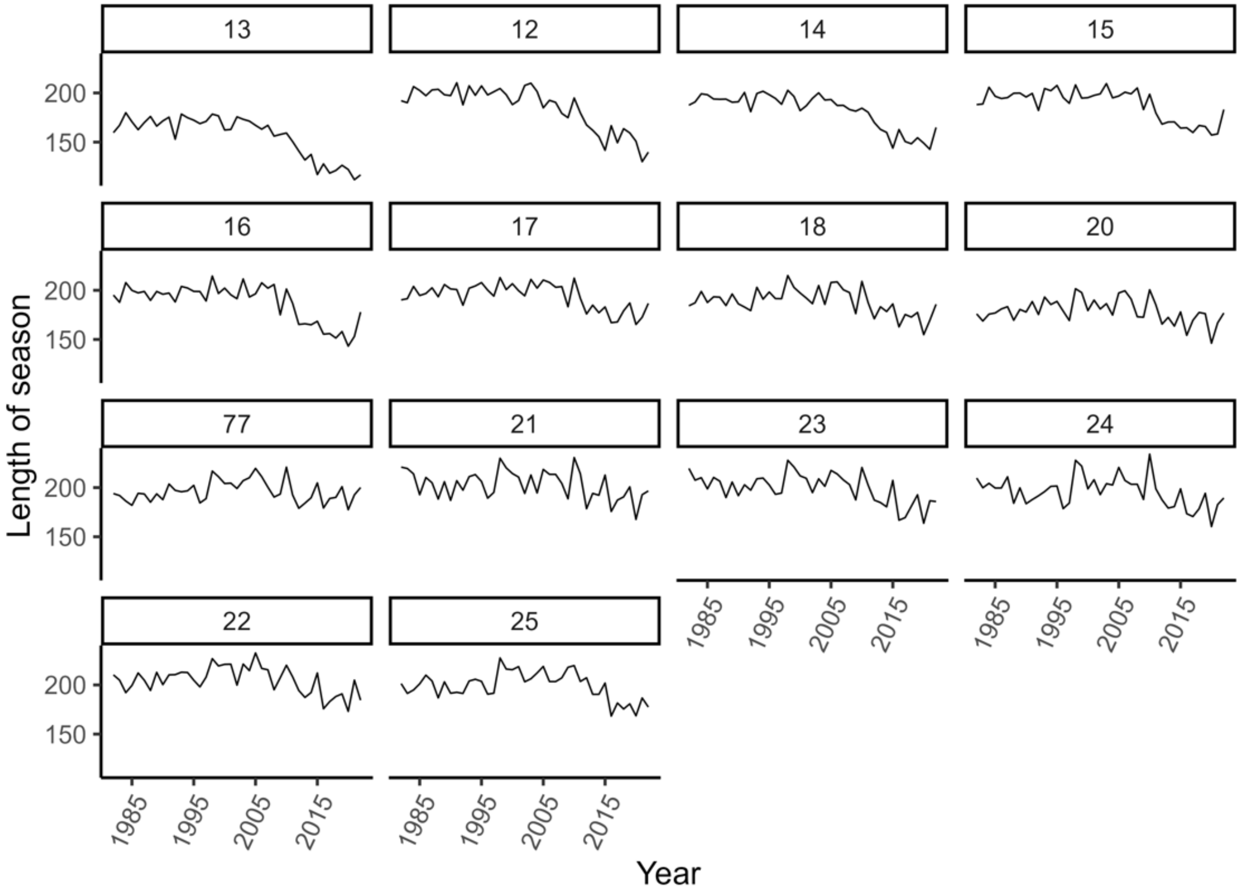

Figure S5) Time series of growing season productivity 1982 to 2019, inferred from the normalized difference vegetation index (NDVI) for each duck survey stratum in the western boreal forest of Canada. Productivity was calculated as the area under the modeled seasonal NDVI curve in the region between the lower and upper 25% thresholds of the seasonal curve, and standardized to mean = 0, SD = 1. Panel labels correspond to stratum names shown in Figure 1 and are arranged in order of decreasing mean latitude.

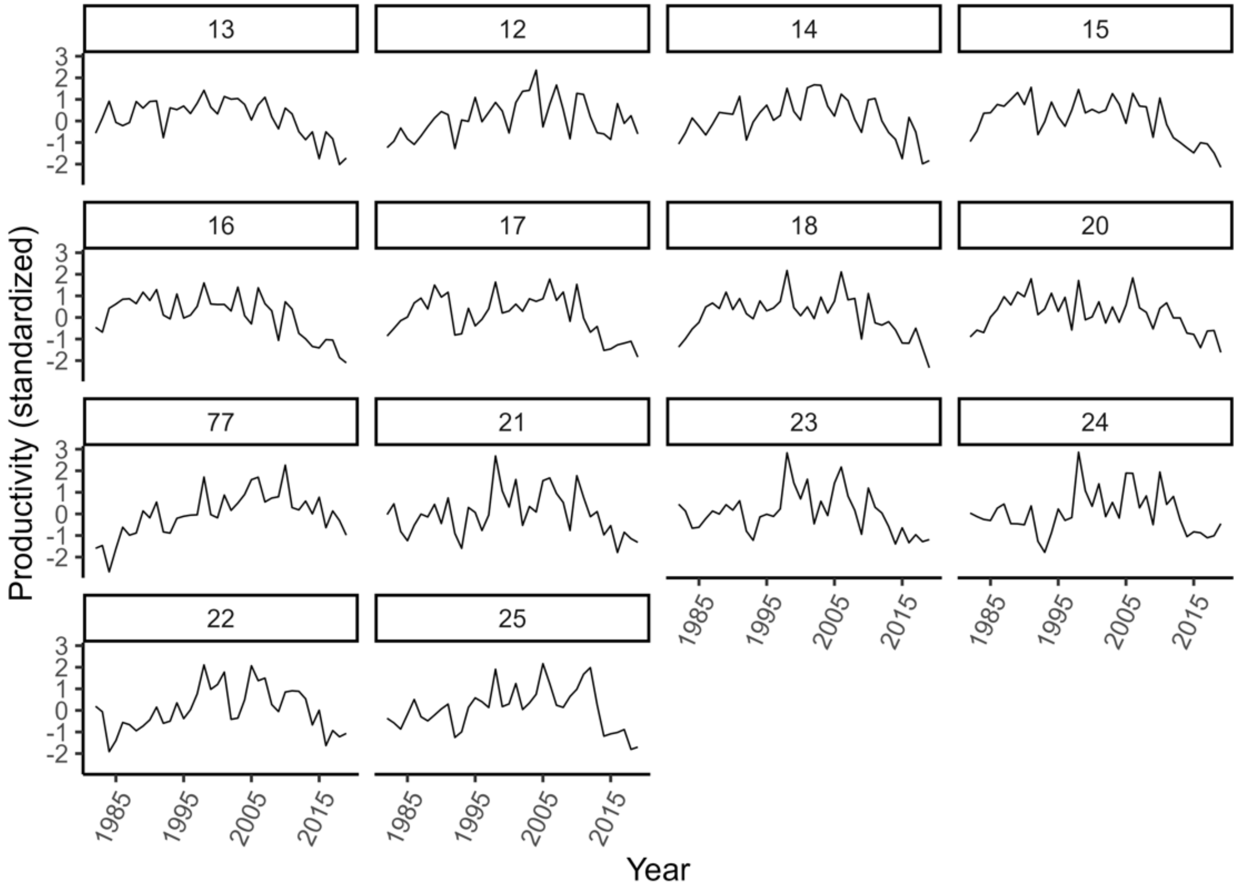

Supplement: Supplementary file 1 — Supplementary file1 (PDF 1791 KB) [file 442_2026_5865_MOESM1_ESM.pdf]
